# Comparison of the copy-neutral loss of heterozygosity identified from whole-exome sequencing data using three different tools

**DOI:** 10.5808/gi.21066

**Published:** 2022-03-31

**Authors:** Gang-Taik Lee, Yeun-Jun Chung

**Affiliations:** 1 Department of Biomedicine & Health Sciences, Graduate School, The Catholic University of Korea, Seoul 06591, Korea; 2 Precision Medicine Research Center, Integrated Research Center for Genome Polymorphism, College of Medicine, The Catholic University of Korea, Seoul 06591, Korea; 3 Department of Microbiology, College of Medicine, The Catholic University of Korea, Seoul 06591, Korea

**Keywords:** CN-LOH, loss of heterozygosity, SNP array, whole-exome sequencing

## Abstract

Loss of heterozygosity (LOH) is a genomic aberration. In some cases, LOH can be generated without changing the copy number, which is called copy-neutral LOH (CN-LOH). CN-LOH frequently occurs in various human diseases, including cancer. However, the biological and clinical implications of CN-LOH for human diseases have not been well studied. In this study, we compared the performance of CN-LOH determination using three commonly used tools. For an objective comparison, we analyzed CN-LOH profiles from single-nucleotide polymorphism array data from 10 colon adenocarcinoma patients, which were used as the reference for comparison with the CN-LOHs obtained through whole-exome sequencing (WES) data of the same patients using three different analysis tools (FACETS, Nexus, and Sequenza). The majority of the CN-LOHs identified from the WES data were consistent with the reference data. However, some of the CN-LOHs identified from the WES data were not consistent between the three tools, and the consistency with the reference CN-LOH profile was also different. The Jaccard index of the CN-LOHs using FACETS (0.84 ± 0.29; mean value, 0.73) was significantly higher than that of Nexus (0.55 ± 0.29; mean value, 0.50; p = 0.02) or Sequenza (0 ± 0.41; mean value, 0.34; p = 0.04). FACETS showed the highest area under the curve value. Taken together, of the three CN-LOH analysis tools, FACETS showed the best performance in identifying CN-LOHs from The Cancer Genome Atlas colon adenocarcinoma WES data. Our results will be helpful in exploring the biological or clinical implications of CN-LOH for human diseases.

## Introduction

Loss of heterozygosity (LOH) is a large-scale genomic aberration, in which one parental allele is lost, resulting in the loss of the heterozygous state. LOH can sometimes be accompanied by an alteration of the copy number; however, most LOH events occur without changing the copy number. This is called copy-neutral LOH (CN-LOH), and it is caused by diverse genetic events such as uniparental disomy or gene conversion [1-3]. CN-LOH has effects on many diseases, such as cardiovascular disease [4], Prader-Willi syndrome [5], and congenital adrenal hyperplasia [6]. In particular, many studies have shown that CN-LOH is a widespread event in diverse cancers, with regular patterns appearing on specific chromosomes [7,8]. The pathogenesis of CN-LOH in carcinogenesis is primarily derived from the biallelic inactivation of a tumor suppressor gene (TSG) [9]. For example, when a TSG in a chromosomal region harbors an inactivating mutation at birth in one allele, this TSG can be completely inactivated by CN-LOH as a second hit, which may induce malignant disease [10,11]. Loss of the wild-type allele of the *KIT* gene and duplication of gain of function mutations in oncogenes due to CN-LOH may also cause tumorigenesis [12]. Despite its potential importance, the biological and clinical implications of CN-LOH on human diseases have not been well studied.

Next-generation sequencing (NGS) technology and recent efforts to collect information on genomic alterations in cancers, such as The Cancer Genome Atlas (TCGA) and COSMIC databases, have facilitated the analysis of CN-LOH in addition to somatic mutations and alteration of gene expression [13]. Several analytical tools have been developed to detect CN-LOH events, using NGS data, such as Sequenza [14], Nexus Copy Number, and FACETS [15]. However, no study has compared the performance of these tools for identifying CN-LOH from whole-exome sequencing (WES) data, which are the most common NGS data for various diseases, including cancer. In this study, we compared the performance of CN-LOH determination by the three commonly used tools.

## Methods

### Data

We used 443 colon adenocarcinoma WES data entries from the TCGA database. Before CN-LOH analysis, the purity and ploidy of each tumor sample were checked using FACETS. To ensure a reliable analysis of CN-LOH, we selected samples with an estimated ploidy of 1.5‒2.6 and an estimated purity above 0.8. Of the tumor samples that fit these criteria, we randomly selected 10 samples for the CN-LOH analysis in this study (Supplementary Tables 1 and 2). We also used Affymetrix SNP Array 6.0 data (CEL files) of the same samples, which were obtained from the GDC Legacy Archive (https://portal.gdc.cancer.gov/legacy-archive/) (Supplementary Table 2). Tumor cell purity and ploidy information of the 10 samples are available in Supplementary Table 3.

### Analysis of CN-LOH using the SNP array data

We first defined the CN-LOH profiles of the 10 colon adenocarcinoma samples using the SNP array data as a reference for the CN-LOH status of the 10 samples. Nexus Copy Number (Biodiscovery Inc., El Segundo, CA, USA) was used to define the LOH and copy number alteration profiles of each sample. The SNP-FASST2 segmentation algorithm was used for copy number estimation with the following parameter values: the minimum number of probes per segment was defined as 5, and the segments showing copy number differences of more than ±0.18 in the log2 scale were defined as copy gain and loss, respectively, as described elsewhere [16]. To obtain CN-LOH, we excluded instances of LOH due to copy number alteration or allelic imbalance. The CN-LOH reference data were converted from hg19 to hg38 using the LiftOver tool (https://genome.ucsc.edu/cgi-bin/hgLiftOver) to match the genome reference with the WES data.

### Analysis of CN-LOH using WES data with different tools

We defined the CN-LOH profiles of the 10 colon adenocarcinoma samples using their WES data (.bam files). Three CN-LOH analysis tools were used: Sequenza, Nexus Copy Number (hereinafter called Nexus), and FACETS. For Sequenza, we analyzed the data by referring to the default setting values following the manufacturer’s instructions. For Nexus, B-allele frequency (BAF) values were defined using the BAM (ngCGH) algorithm, and copy number estimation was performed using the SNPRank segmentation algorithm following the manufacturer’s instructions. All other conditions were implemented according to the default values provided by the manufacturer. CN-LOH analysis using FACETS was performed using the default values. Of the LOHs identified from the WES analysis, we removed the regions of allelic imbalance and copy number aberrations by using the Nexus filtering option to ensure the identification of CN-LOHs. To compare the CN-LOH calls between the reference and the three tools, we used a log-R ratio (LRR) plot and BAF plot using “KaryoploteR” [17]. The probe intensity values and SNP intensity values of the reference, which were required for the LRR and BAF plots, were calculated using Nexus. In detail, the accuracy of CN-LOH calls using the three tools was estimated based on the overlapping of CN-LOH calls between each tool and the reference. If a CN-LOH region deduced from the tool overlapped with the CN-LOH region in the reference set, we defined this call as consistent with the reference regardless of the length of the overlapping region.

### Statistical analysis

To estimate the similarity between the reference data and the analysis data using the three tools, we used the Jaccard index. The equation is as follows:


$$\text{Jaccard Index}=\frac{\text{n(Reference Result ∩ Tool Result)}}{\text{n(Reference Result ∪ Tool Result)}}=\text{J(Tool)}$$

We also used the “ROCR” [18] R package to calculate the area under the curve (AUC), sensitivity, and specificity for each tool.

## Results

**Fig. 1.**
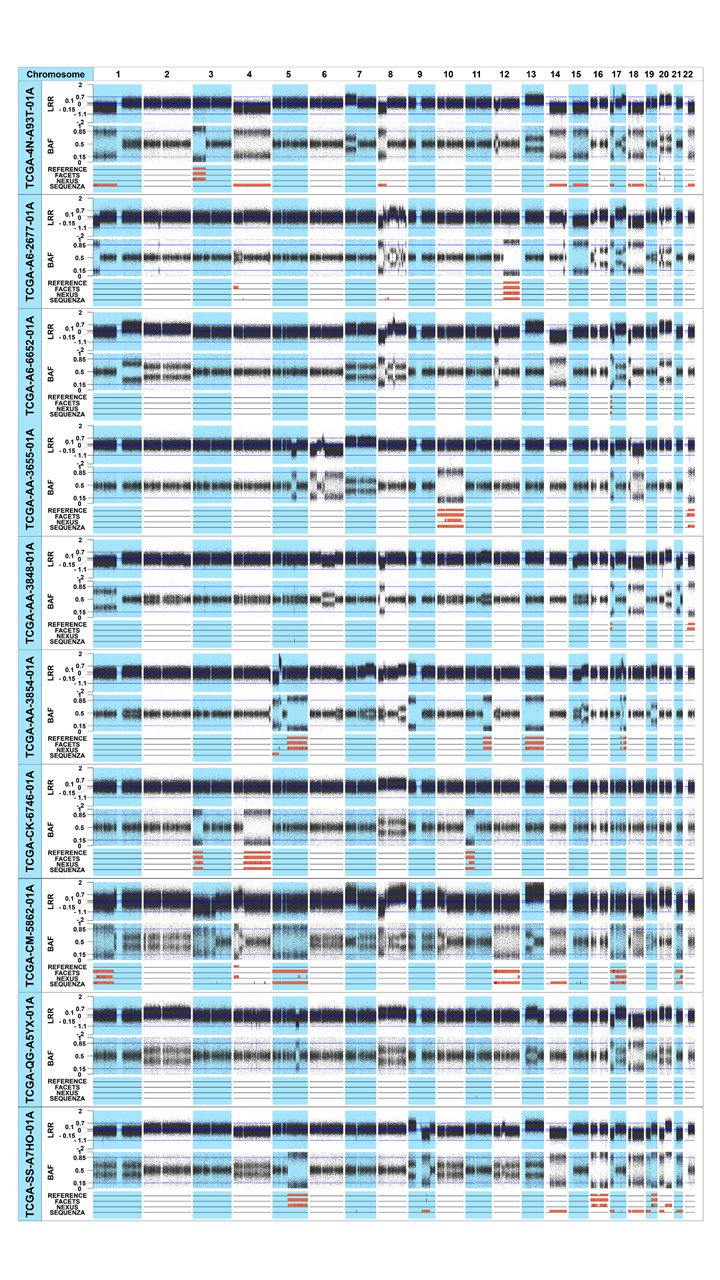
CN-LOHs identified from the 10 colon adenocarcinoma patients. The upper, middle, and lower plots of each sample represent the LRR, BAF plot, and the CN-LOHs identified from each tool, respectively. In the lower plot, reference CN-LOHs were defined from the SNP array data of the 10 colon adenocarcinoma patients. The CN-LOHs of the three tools were defined from the TCGA WES data of the same patients. The orange regions represent the CN-LOH area. CN-LOH, copy-neutral loss of heterogeneity; LRR, log-R ratio plot; BAF, B-allele frequency; SNP, single-nucleotide polymorphism; TCGA, The Cancer Genome Atlas; WES, whole-exome sequencing.

We analyzed the CN-LOH profiles using the SNP array data of the 10 colon adenocarcinoma patients. In total, 25 CN-LOH events were identified from the 10 patients according to the reference data (Table 1, Fig. 1). The average number of the reference CN-LOH events identified from the SNP array data was 2.5 per sample (range, 0 to 6), the average length was 44.5 Mb (range, 1.5 to 136.4 Mb), and the median length was 30 Mb.

We next analyzed the CN-LOH profiles of the WES data from the 10 colon adenocarcinomas using the three analysis tools (Nexus, FACETS, and Sequenza) (Table 1, Fig. 1). The average number of CN-LOH events identified from the TCGA WES data was 4.7 (range, 0 to 17), 10.3 (range, 0 to 25), and 11.1 (range, 1 to 41) for FACETS, Nexus, and Sequenza, respectively. The average size of the CN-LOH events was 35.3 Mb, 10.9 Mb, and 18.9 Mb according to FACETS, Nexus, and Sequenza, respectively. The details of the CN-LOH profiles of the 10 samples using the WES data with the three analysis tools are available in Supplementary Table 4.

**Fig. 2.**
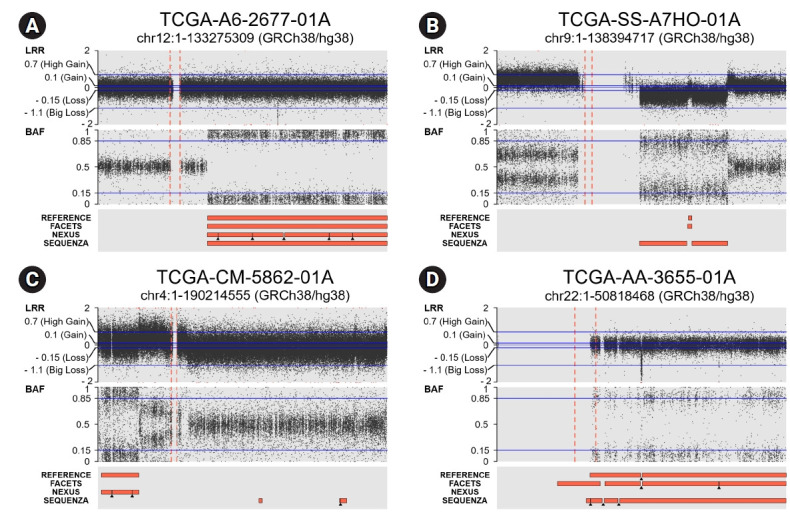
(A) A CN-LOH on 12q (50,510,007–133,265,309 bp) identified from reference (SNP array data) was consistently identified from the WES data by all three tools. (B) A CN-LOH (91,605,233–93,165,795 bp) within the copy loss area on 9q identified from reference was consistently identified by FACETS, but not by Nexus or Sequenza. (C) A CN-LOH on 4p was identified by reference and Nexus, but not by FACETS and Sequenza. (D) CN-LOH events on chromosome 22 were identified by reference, FACETS, and Sequenza, however, Sequenza could not discriminate the small sized homozygous deletion (25,662,836–25,920,518 bp) within the CN-LOH region, which was detected by reference and FACETS. CN-LOH, copy-neutral loss of heterogeneity; SNP, single nucleotide polymorphism; WES, whole-exome sequencing; LRR, log-R ratio plot; BAF, B-allele frequency.

Most CN-LOHs identified from TCGA WES data using the three tools were consistent with the reference data identified from SNP array data. Of the 25 reference CN-LOHs, 24 (96%) were consistently detected by FACETS. The consistency of the CN-LOHs identified by Nexus and Sequenza with the reference profile was lower than that of FACETS: 76% (19/25) for Nexus and 28% (7/25) for Sequenza, respectively. Examples of CN-LOHs consistently or inconsistently defined between the reference data and the three tools are illustrated in Fig. 2. In the TCGA-A6-2677-01A sample, according the LRR plot from the SNP array data, there was no copy number change in chromosome 12. There was a CN-LOH in the q-arm (chr12: 50,510,007‒133,265,309 bp), which all three tools consistently identified from the WES data (Fig. 2A). Some of the CN-LOHs identified from the TCGA WES data were not consistent between the three tools, and the consistency with the reference CN-LOH profile was also different. In the TCGA-SS-A7HO-01A sample (Fig. 2B), there was a copy loss in the q-arm of chromosome 9, and a small (91,605,233‒93,165,795 bp) CN-LOH event occurred inside this copy loss area. This CN-LOH event identified by SNP array data was consistently detected by FACETS, but was not identified by Nexus or Sequenza. In the TCGA-CM-5862-01A sample (Fig. 2C), the reference and Nexus identified a CN-LOH event on chromosome 4, but FACETS and Sequenza did not. In the TCGA-AA-3655-01A sample (Fig. 2D), the reference and FACETS detected two CN-LOH events on chromosome 22, and Sequenza also detected these CN-LOHs; however, Sequenza could not discriminate the small homozygous deletion in this region (25,662,836‒25,920,518 bp). Nexus could not identify the CN-LOHs in this case.

**Fig. 3.**
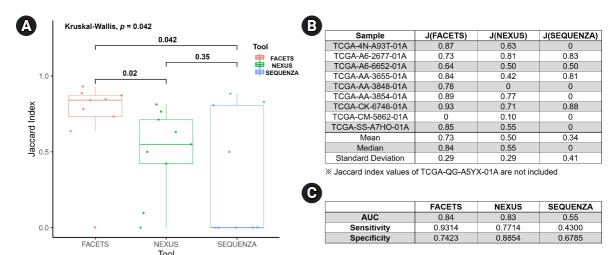
Comparison of the CN-LOH analysis tools. (A) Box plot of the Jaccard index for the three tools, with p-values are provided above the brackets. (B) Jaccard index table for each sample analyzed using the three tools. (C) ROC analyses for the three tools. CN-LOH, copy-neutral loss of heterogeneity; ROC, receiver operating characteristic.

For an objective comparison of the CN-LOH analysis tools, we analyzed the Jaccard index, which represents the similarity of CN-LOH calls between the tools and the reference data, as described in the Methods section. The median value of the Jaccard index of FACETS (0.84 ± 0.29; mean value, 0.73) was significantly higher than those of Nexus (0.55 ± 0.29; mean value, 0.50; p = 0.02) and Sequenza (0 ± 0.41; mean value, 0.34; p = 0.04) (Fig. 3A and 3B). Nexus showed a better Jaccard index than Sequenza, but the difference was not statistically significant. As another method of verification, we performed receiver operating characteristic analyses for the three tools. In the receiver operating characteristic analysis, FACETS showed the best identification power (AUC, 0.84; sensitivity, 0.9314; specificity, 0.7423) compared with Nexus (AUC, 0.83; sensitivity, 0.7714; specificity, 0.8854) and Sequenza (AUC, 0.55; sensitivity, 0.4300; specificity, 0.6785) (Fig. 3C). Collectively, these results suggest that FACETS may be the most suitable tool for identifying CN-LOHs from WES data.

**Fig. 4.**
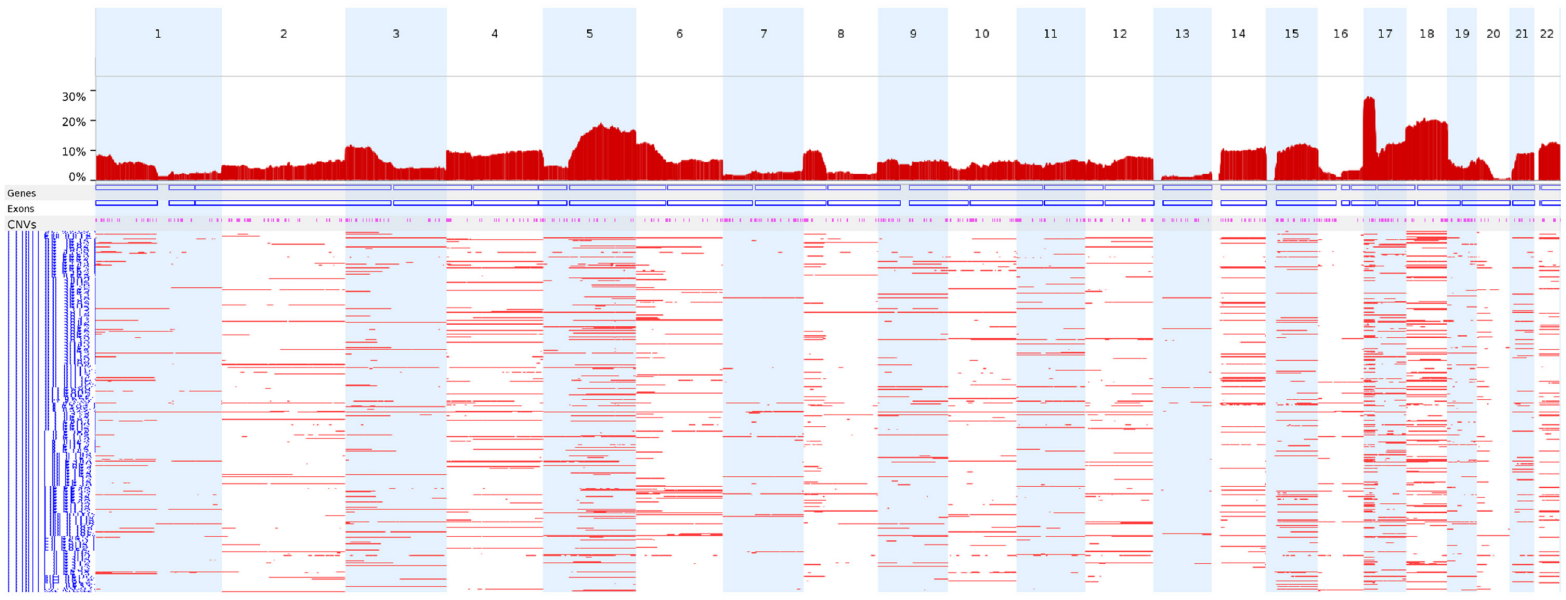
The genome-wide frequencies of CN-LOHs in the TCGA colon adenocarcinomas. TCGA colon adenocarcinoma WES data (n = 398) were analyzed using FACETS under the conditions used in this study to identify CN-LOHs. CN-LOH, copy-neutral loss of heterogeneity; TCGA, The Cancer Genome Atlas; WES, whole-exome sequencing.

We next analyzed the CN-LOH profiles of the 398 TCGA colon adenocarcinoma WES data entries using FACETS under the conditions used in this study to identify CN-LOH. Chromosome 17p (17p13.1) showed the highest frequency of CN-LOHs (28.1%), followed by chromosomes 18 and 5q (Fig. 4).

## Discussion

It is well known that copy number alterations can induce over-activation or inactivation of cancer-related genes, which consequently contribute to tumorigenesis or the progression of cancer. However, the biological implications of CN-LOH for tumorigenesis have not been well studied. A reason for this is the difficulties in identifying CN-LOH, especially with WES data. Although several algorithms have been developed, there is no global standard method for the CN-LOH analysis using WES data. To detect genome-wide CN-LOHs, WES data would be relatively disadvantageous because the exonic region is a small part of the whole genome. However, despite this disadvantage, CN-LOH identification from WES data would be valuable for understanding the pathogenesis of diseases, because WES is a common source of data for diverse diseases, including cancer.

For an objective comparison of the CN-LOH identification performance of the three tools, we used TCGA colon adenocarcinoma WES data because Affymetrix SNP Array 6.0 data of the same samples were available in the GDC Legacy Archive (https://portal.gdc.cancer.gov/legacy-archive/). To verify instances of CN-LOH, we selected samples with an estimated ploidy of 1.5-2.6, which suggests that the majority of the genome would have a copy-neutral status. Furthermore, to minimize the effect of normal cell contamination, we selected samples with an estimated purity >0.8. For an objective comparison of the tools, we used CN-LOHs from the SNP array data of the same colon adenocarcinomas as reference data.

FACETS showed greater consistency of CN-LOH calls with the reference data than Nexus and Sequenza. Accordingly, the Jaccard index of FACETS was significantly higher than those of Nexus and Sequenza. The Jaccard index is a coefficient that shows the degree of similarity between different datasets [19]; therefore, a higher Jaccard index (converging to 1) indicates greater similarity between different groups. Taken together, our data suggest that FACETS would be suitable to identify CN-LOHs from WES data.

When we applied this condition to the TCGA colon adenocarcinoma WES data, chromosomes 17p, 18, and 5q showed frequent CN-LOH events, which is largely consistent with previous reports of the CN-LOH profiles in colon adenocarcinomas using 63 sets of whole-genome sequencing data [20].

There are several limitations of this study. First, we used the default settings of the tools, which might not have been the best conditions to obtain maximally reliable CN-LOH profiles. Second, we did not explore the reasons for the inconsistency of CN-LOH calls between the three tools in this study. Third, the quality of the WES data was most likely different among the 10 samples, which may have affected the reliability of CN-LOH calling.

In conclusion, of the three CN-LOH analysis tools, FACETS showed the best performance in identifying CN-LOHs from TCGA colon adenocarcinoma WES data. Our results may be helpful for exploring the biological or clinical implications of CN-LOH for human diseases.

## Figures and Tables

**Table 1.** CN-LOHs identified from the 10 colon adenocarcinoma patients

	Reference	FACETS	Nexus	Sequenza
Samples				
TCGA-4N-A93T-01A	2	3	11	22
TCGA-A6-2677-01A	1	2	6	5
TCGA-A6-6652-01A	1	1	1	3
TCGA-AA-3655-01A	4	5	3	11
TCGA-AA-3848-01A	2	2	-	1
TCGA-AA-3854-01A	5	7	21	2
TCGA-CK-6746-01A	3	4	14	11
TCGA-CM-5862-01A	1	17	22	41
TCGA-QG-A5YX-01A	-	-	-	1
TCGA-SS-A7HO-01A	6	6	25	14
Total No. of CN-LOH	25	47	103	111
Average No. of CN-LOH	2.5	4.7	10.3	11.1
Minimal length (bp)	1,560,563	704,899	2,028,231	7,655
Maximal length (bp)	136,468,365	181,230,348	71,438,575	137,587,418
Mean length (bp)	44,481,811	35,328,191	10,864,090	18,883,527
Median length (bp)	30,037,847	15,273,966	7,929,436	4,336,034

CN-LOH, copy-neutral loss of heterogeneity; TCGA, The Cancer Genome Atlas.

## Supplementary Materials

Supplementary Table 1. List of TCGA-COAD WES data files of the 10 colon adenocarcinomas used in this study

Supplementary Table 2. List of TCGA-COAD SNP array data files of the 10 colon adenocarcinomas used in this study

Supplementary Table 3. Tumor cell purity and ploidy information of the 10 colon adenocarcinomas

Supplementary Table 4. Number and characteristics of the CN-LOHs identified by the three tools
