# Supplementary material for: Comparison of the copy-neutral loss of heterozygosity identified from whole-exome sequencing data using three different tools
Source: Genomics Inform. 2022 Mar 31;20(1):e4. doi: 10.5808/gi.21066 (PMC9001996; doi:10.5808/gi.21066)
Supplement: Supplementary Table 1. — List of TCGA-COAD WES data files of the 10 colon adenocarcinomas used in this study [file gi-21066suppl1.pdf]

Supplementary Table 1. List of TCGA-COAD WES data files of the 10 colon adenocarcinomas used in this study

Sample list	WES file for normal	WES file for tumor
TCGA-4N-A93T-01A	TCGA-4N-A93T-10A-01D-A370-10_Illumina_gdc_realn.bam	TCGA-4N-A93T-01A-11D-A36X-10_Illumina_gdc_realn.bam
TCGA-A6-2677-01A	TCGA-A6-2677-01A-01D-A270-10_Illumina_gdc_realn.bam	TCGA-A6-2677-01A-01D-A270-10_Illumina_gdc_realn.bam
TCGA-A6-6652-01A	TCGA-A6-6652-10A-01D-1771-10_hg19_Illumina_gdc_realn.bam	TCGA-A6-6652-01A-11D-1771-10_hg19_Illumina_gdc_realn.bam
TCGA-AA-3655-01A	TCGA-AA-3655-11A-01D-1719-10_hg19_Illumina_gdc_realn.bam	TCGA-AA-3655-01A-02D-1719-10_hg19_Illumina_gdc_realn.bam
TCGA-AA-3848-01A	47b245656a2fae085cb620e47270447b_gdc_realn.bam	fbacff30e37deabcea42c761510057dd_gdc_realn.bam
TCGA-AA-3854-01A	d583aaa2918b4e0518c8dc1b6a947798_gdc_realn.bam	9e47d76983d3f18b9037686de9d05c7a_gdc_realn.bam
TCGA-CK-6746-01A	TCGA-CK-6746-10A-01D-1835-10_Illumina_gdc_realn.ba	TCGA-CK-6746-01A-11D-1835-10_Illumina_gdc_realn.bam
TCGA-CM-5862-01A	TCGA-CM-5862-10A-01D-1650-10_hg19_Illumina_gdc_realn.bam	TCGA-CM-5862-01A-01D-1650-10_hg19_Illumina_gdc_realn.bam
TCGA-QG-A5YX-01A	TCGA-QG-A5YX-10A-01D-A28G-10_Illumina_gdc_realn.bam	TCGA-QG-A5YX-01A-11D-A28G-10_Illumina_gdc_realn.bam
TCGA-SS-A7HO-01A	TCGA-SS-A7HO-10A-01D-A370-10_Illumina_gdc_realn.bam	TCGA-SS-A7HO-01A-21D-A36X-10_Illumina_gdc_realn.bam

TCGA, The Cancer Genome Atlas; COAD, colon adenocarcinoma; WES, whole-exome sequencing.
