# Supplementary material for: Comparison of the copy-neutral loss of heterozygosity identified from whole-exome sequencing data using three different tools
Source: Genomics Inform. 2022 Mar 31;20(1):e4. doi: 10.5808/gi.21066 (PMC9001996; doi:10.5808/gi.21066)
Supplement: Supplementary Table 2. — List of TCGA-COAD SNP array data files of the 10 colon adenocarcinomas used in this study [file gi-21066suppl2.pdf]

Supplementary Table 2. List of TCGA-COAD SNP array data files of the 10 colon adenocarcinomas used in this study

Sample list	SNP array file for normal	Tumor array file
TCGA-4N-A93T-01A	DADOS_p_TCGAb3_85_86_87_88_NSP_GenomeWideSNP_6_B08_1464750.CEL	DADOS_p_TCGAb3_85_86_87_88_NSP_GenomeWideSNP_6_B09_1464716.CEL
TCGA-A6-2677-01A	VENUE_p_TCGAb28_SNP_N_GenomeWideSNP_6_C02_568944.CEL	VENUE_p_TCGAb28_SNP_N_GenomeWideSNP_6_E08_568968.CEL
TCGA-A6-6652-01A	KNELT_p_TCGA_b123_131_SNP_N_GenomeWideSNP_6_D07_787752.CEL	KNELT_p_TCGA_b123_131_SNP_N_GenomeWideSNP_6_A09_787772.CEL
TCGA-AA-3655-01A	GRIPS_p_TCGA_b116_SNP_N_GenomeWideSNP_6_F01_781390.CEL	GRIPS_p_TCGA_b116_SNP_N_GenomeWideSNP_6_F02_781446.CEL
TCGA-AA-3848-01A	SONGS_p_TCGAb36_SNP_N_GenomeWideSNP_6_F05_585296.CEL	SONGS_p_TCGAb36_SNP_N_GenomeWideSNP_6_G11_585344.CEL
TCGA-AA-3854-01A	SONGS_p_TCGAb36_SNP_N_GenomeWideSNP_6_D04_585408.CEL	SONGS_p_TCGAb36_SNP_N_GenomeWideSNP_6_G04_585364.CEL
TCGA-CK-6746-01A	BAIZE_p_TCGA_b138_SNP_N_GenomeWideSNP_6_A11_808856.CEL	BAIZE_p_TCGA_b138_SNP_N_GenomeWideSNP_6_H10_808770.CEL
TCGA-CM-5862-01A	SNORT_p_TCGA_b89_SNP_N_GenomeWideSNP_6_E11_777348.CEL	SNORT_p_TCGA_b89_SNP_N_GenomeWideSNP_6_A06_777302.CEL
TCGA-QG-A5YX-01A	LEGIT_p_TCGA_300_301_302_N_GenomeWideSNP_6_A01_1344376.CEL	LEGIT_p_TCGA_300_301_302_N_GenomeWideSNP_6_A02_1344314.CEL
TCGA-SS-A7HO-01A	DADOS_p_TCGAb3_85_86_87_88_NSP_GenomeWideSNP_6_A11_1464674.CEL	DADOS_p_TCGAb3_85_86_87_88_NSP_GenomeWideSNP_6_A10_1464744.CEL

TCGA, The Cancer Genome Atlas; COAD, colon adenocarcinoma; SNP, single nucleotide polymorphism.
