# Supplementary material for: Comparison of the copy-neutral loss of heterozygosity identified from whole-exome sequencing data using three different tools
Source: Genomics Inform. 2022 Mar 31;20(1):e4. doi: 10.5808/gi.21066 (PMC9001996; doi:10.5808/gi.21066)
Supplement: Supplementary Table 3. — Tumor cell purity and ploidy information of the 10 colon adenocarcinomas [file gi-21066suppl3.pdf]

Supplementary Table 3. Tumor cell purity and ploidy information of the 10 colon adenocarcinomas

Sample list	Purity	Ploidy
TCGA-4N-A93T-01A	0.82	1.83
TCGA-A6-2677-01A	0.85	2.05
TCGA-A6-6652-01A	0.80	2.38
TCGA-AA-3655-01A	0.80	2.06
TCGA-AA-3848-01A	0.82	1.97
TCGA-AA-3854-01A	0.86	2.21
TCGA-CK-6746-01A	0.82	2.05
TCGA-CM-5862-01A	0.85	2.55
TCGA-QG-A5YX-01A	0.80	2.13
TCGA-SS-A7HO-01A	0.87	1.85
