# Supplementary material for: Comparison of the copy-neutral loss of heterozygosity identified from whole-exome sequencing data using three different tools
Source: Genomics Inform. 2022 Mar 31;20(1):e4. doi: 10.5808/gi.21066 (PMC9001996; doi:10.5808/gi.21066)
Supplement: Supplementary Table 4. — Number and characteristics of the CN-LOHs identified by the three tools [file gi-21066suppl4.pdf]

Supplementary Table 4. Number and characteristics of the CN-LOHs identified by the three tools

	chr 1	chr 2	chr 3	chr 4	chr 5	chr 6	chr 7	chr 8	chr 9	chr 10	chr 11	chr 12	chr 13	chr 14	chr 15	chr 16	chr 17	chr 18	chr 19	chr 20	chr 21	chr 22	**Total**	**Minimal Length (bp)**	**Maximal Length (bp)**	**Mean Length (bp)**	**Median Length (bp)**
**TCGA-4N-A93T-01A**																											
REFERENCE	0	0	1	0	0	0	0	0	0	0	0	0	0	0	0	0	0	0	0	1	0	0	2	4,221,855	65,996,955	35,109,405	35,109,405
FACETS	0	0	1	0	0	0	0	0	0	0	0	0	0	0	0	0	0	0	0	2	0	0	3	1,491,123	65,718,092	23,393,565	2,971,481
NEXUS	0	0	9	0	0	0	0	0	0	0	0	0	0	0	0	0	0	0	0	2	0	0	11	2,030,946	15,756,204	6,356,533	5,420,390
SEQUENZA	5	0	0	2	0	0	0	3	0	0	0	0	0	4	1	0	2	2	2	0	0	1	22	505,344	137,587,418	29,475,257	14,225,795
**TCGA-A6-2677-01A**																											
REFERENCE	0	0	0	0	0	0	0	0	0	0	0	1	0	0	0	0	0	0	0	0	0	0	1	-	82,755,303	-	-
FACETS	0	0	0	1	0	0	0	0	0	0	0	1	0	0	0	0	0	0	0	0	0	0	2	24,977,259	82,773,682	53,875,471	53,875,471
NEXUS	0	0	0	0	0	0	0	0	0	0	0	6	0	0	0	0	0	0	0	0	0	0	6	4,866,925	20,439,669	13,685,961	15,017,510
SEQUENZA	0	0	0	2	0	0	0	2	0	0	0	1	0	0	0	0	0	0	0	0	0	0	5	222,238	82,771,762	18,408,407	1,067,777
**TCGA-A6-6652-01A**																											
REFERENCE	0	0	0	0	0	0	0	0	0	0	0	0	0	0	0	0	1	0	0	0	0	0	1	-	8,446,453	-	-
FACETS	0	0	0	0	0	0	0	0	0	0	0	0	0	0	0	0	1	0	0	0	0	0	1	-	8,396,063	-	-
NEXUS	0	0	0	0	0	0	0	0	0	0	0	0	0	0	0	0	1	0	0	0	0	0	1	-	10,445,531	-	-
SEQUENZA	0	0	0	0	0	0	0	0	0	0	0	0	1	0	0	0	2	0	0	0	0	0	3	52,471	8,973,810	3,147,664	416,712
**TCGA-AA-3655-01A**																											
REFERENCE	0	0	0	0	0	0	0	0	0	2	0	0	0	0	0	0	0	0	0	0	0	2	4	8,899,680	90,896,548	40,552,685	31,207,255
FACETS	0	0	0	0	0	0	0	0	0	1	0	0	0	0	0	0	0	0	0	0	0	4	5	6,248,912	133,642,256	34,509,924	11,767,568
NEXUS	0	0	0	0	0	0	0	0	0	3	0	0	0	0	0	0	0	0	0	0	0	0	3	2,610,614	71,438,575	26,082,715	4,198,956
SEQUENZA	0	0	0	0	0	0	0	0	0	7	0	0	0	0	0	0	0	0	0	0	0	4	11	359,090	86,995,177	14,955,404	3,972,794
**TCGA-AA-3848-01A**																											
REFERENCE	0	0	0	0	0	0	0	0	0	0	0	0	0	0	0	0	1	0	0	0	0	1	2	11,025,390	33,764,742	22,395,066	22,395,066
FACETS	0	0	0	0	0	0	0	0	0	0	0	0	0	0	0	0	1	0	0	0	0	1	2	10,114,644	38,178,375	24,146,510	24,146,510

NEXUS	0	0	0	0	0	0	0	0	0	0	0	0	0	0	0	0	0	0	0	0	0	0	0	-	-	-	-
SEQUENZA	0	0	0	0	1	0	0	0	0	0	0	0	0	0	0	0	0	0	0	0	0	0	1	718,136	34,187,128	17,452,632	17,452,632
**TCGA-AA-3854-01A**																											
REFERENCE	0	0	0	0	1	0	0	0	0	0	1	0	1	0	0	0	2	0	0	0	0	0	5	7,232,501	105,813,523	53,560,768	43,678,897
FACETS	0	0	0	0	3	0	0	0	0	0	1	0	1	0	0	0	2	0	0	0	0	0	7	704,899	96,152,761	37,152,734	33,550,386
NEXUS	0	0	0	0	11	0	0	0	0	0	2	0	4	0	0	0	4	0	0	0	0	0	21	2,028,231	38,063,814	12,422,872	9,413,860
SEQUENZA	0	0	0	0	2	0	0	0	0	0	0	0	0	0	0	0	0	0	0	0	0	0	2	580,934	31,662,627	16,121,781	16,121,781
**TCGA-CK-6746-01A**																											
REFERENCE	0	0	1	1	0	0	0	0	0	0	1	0	0	0	0	0	0	0	0	0	0	0	3	47,542,805	136,468,365	78,652,761	51,947,112
FACETS	0	0	1	2	0	0	0	0	0	0	1	0	0	0	0	0	0	0	0	0	0	0	4	15,865,830	121,314,641	58,648,065	48,705,895
NEXUS	0	0	2	10	0	0	0	0	0	0	2	0	0	0	0	0	0	0	0	0	0	0	14	3,729,588	45,593,759	14,616,653	11,975,517
SEQUENZA	0	0	2	5	0	0	0	0	0	0	3	0	0	0	1	0	0	0	0	0	0	0	11	15,898	77,352,994	21,463,820	17,092,685
**TCGA-CM-5862-01A**																											
REFERENCE	0	0	0	1	0	0	0	0	0	0	0	0	0	0	0	0	0	0	0	0	0	0	1	-	24,627,482	-	-
FACETS	3	0	0	0	1	0	0	0	0	0	0	6	0	0	0	0	6	0	0	0	1	0	17	1,531,345	181,230,348	31,354,052	14,830,299
NEXUS	9	0	0	3	2	0	0	0	0	0	0	1	0	0	0	0	4	0	0	0	3	0	22	2,641,350	41,481,194	8,812,555	5,152,235
SEQUENZA	4	0	1	3	6	0	0	0	0	0	0	12	0	2	3	0	9	0	0	0	1	0	41	129,446	127,712,980	14,774,448	3,363,870
**TCGA-QG-A5YX-01A**																											
REFERENCE	0	0	0	0	0	0	0	0	0	0	0	0	0	0	0	0	0	0	0	0	0	0	0	-	-	-	-
FACETS	0	0	0	0	0	0	0	0	0	0	0	0	0	0	0	0	0	0	0	0	0	0	0	-	-	-	-
NEXUS	0	0	0	0	0	0	0	0	0	0	0	0	0	0	0	0	0	0	0	0	0	0	0	-	-	-	-
SEQUENZA	0	0	0	0	0	0	0	0	0	0	1	0	0	0	0	0	0	0	0	0	0	0	1	-	1,080,783	-	-
**TCGA-SS-A7HO-01A**																											
REFERENCE	0	0	0	0	1	0	0	0	1	0	0	0	0	0	0	3	0	0	1	0	0	0	6	1,560,563	103,514,263	35,872,374	30,037,847

FACETS	0	0	0	0	1	0	0	0	1	0	0	0	0	0	0	2	0	0	2	0	0	0	6	2,015,009	102,810,582	37,595,728	21,598,415
NEXUS	0	0	0	0	8	0	0	0	0	0	0	0	0	0	0	8	0	0	6	3	0	0	25	2,049,132	34,890,907	8,755,213	5,184,071
SEQUENZA	0	0	0	0	0	0	1	0	2	0	0	0	0	2	0	1	2	2	2	1	1	0	14	7,655	86,921,737	21,837,464	18,362,372

CN-LOH, copy-neutral loss of heterozygosity.
